# Inflammatory status, body composition and ethnic differences in bone mineral density: The Southall and Brent Revisited Study

**DOI:** 10.1016/j.bone.2021.116286

**Published:** 2022-02

**Authors:** Ruth Durdin, Camille Parsons, Elaine M. Dennison, Suzanne Williams, Therese Tillin, Nishi Chaturvedi, Cyrus Cooper, Nicholas C. Harvey, Kate A. Ward

**Affiliations:** a MRC Lifecourse Epidemiology Centre, University of Southampton, Southampton General Hospital, Southampton, UK; b National Institute for Health Research Southampton Biomedical Research Centre, University of Southampton and University Hospital Southampton NHS Foundation Trust, Southampton, UK; c MRC Unit for Lifelong Health and Ageing, University College London, London, UK; d Institute of Musculoskeletal Science, Nuffield Department of Orthopaedics, Rheumatology and Musculoskeletal Science, University of Oxford, Oxford, UK

**Keywords:** Ethnicity, Bone mineral density, Body composition, Inflammation, Osteoporosis, Epidemiology

## Abstract

Ethnic differences in bone mineral density (BMD) and fracture risk are well-described; the aim of this study was to investigate whether central adiposity or inflammatory status contribute to these ethnic differences in BMD in later life.

The Southall and Brent Revisited study (SABRE) is a UK-based tri-ethnic cohort of men and women of European, South Asian or African Caribbean origin. At the most recent SABRE follow-up (2014–2018), in addition to measures of cardiometabolic phenotype, participants had dual-energy X-ray absorptiometry (DXA) bone and body composition scans. Multiple linear regression was used to determine whether markers of body composition, central adiposity or inflammatory status contributed to ethnic differences in BMD.

In men and women, age- and height-adjusted BMD at all sites was higher in African Caribbeans compared to Europeans (femoral neck: standardised β (95% confidence interval): men: 1.00SD (0.75, 1.25); women: 0.77SD (0.56, 0.99)). South Asian men had higher BMD than European men at the hip (femoral neck: 0.34SD (95%CI: 0.15, 0.54)). Although adjustment for body mass index (BMI) or lean mass index (LMI) at the lumbar spine reduced the size of the difference in BMD between African Caribbean and European men (age and height adjusted difference: 0.35SD (0.08, 0.62); age and BMI adjusted difference: 0.25SD (−0.02, 0.51)), in both men and women ethnic differences remained after adjustment for measures of central adiposity (estimated visceral adipose tissue mass (VAT mass) and android to gynoid ratio) and inflammation (interleukin-6 (logIL-6) and C-reactive protein (logCRP)). Furthermore, in women, we observed ethnic differences in the relationship between BMI (overall interaction: *p* = 0.04), LMI (p = 0.04) or VAT mass (*p* = 0.009) and standardised lumbar spine BMD.

In this tri-ethnic cohort, ethnic differences in BMD at the femoral neck, total hip or lumbar spine were not explained by BMI, central adiposity or inflammatory status. Given ethnic differences in fracture incidence, it is important to further investigate why ethnic differences in BMD exist.

## Highlights

• Ethnic differences in BMD in a UK tri-ethnic cohort of men and women exist.
• In women associations between adiposity and spine BMD differed across ethnic groups.
• Ethnic differences in BMD were not explained by BMI, adiposity or inflammation.

## 1 Introduction

Ethnic differences in fracture incidence exist in men and women in the UK, where fracture incidence is highest in White and lowest in Black individuals [1]. Previous studies of ethnic differences in bone mineral density (BMD) generally show a similar pattern with BMD being highest in Afro-Caribbean and African American, and similar in South Asian compared to Caucasian and White, men and women [2], [3], [4], [5]. Whilst these differences are well-described, and clearly there is a strong genetic component to BMD [6], there is a need to further explore the underlying environmental determinants of the differences in BMD, especially as populations age and the risk of fracture increases. Aging is also associated with multimorbidity [7], and there are shared risk factors for non-communicable diseases including cardiometabolic and musculoskeletal health. Therefore, given that there are ethnic differences in cardiometabolic health [8], further investigation of the links between cardiometabolic phenotype and bone health are particularly important as differences in body composition may not only contribute to cardiometabolic disease risk but also bone health.

Ethnic differences in body composition, including differences in fat, muscle mass [9] and visceral adipose tissue (VAT) levels [10], [11] have been previously described. Weight, which includes lean and fat mass, is a source of load on the skeleton. The relationship between fat mass and bone is complex given that, as well as a mechanical loading effect via weight, adipose tissue is also a source of hormones and mediators including estrogen, adipokines and cytokines [12]. Estrogen is a major regulator of bone metabolism and has positive effects [13]; in contrast, inflammatory cytokines may negatively impact bone health [14]. Clinically, some studies show weak or no associations between inflammatory biomarkers and BMD [15] and others demonstrate associations between C-reactive protein (CRP) or interleukin-6 (IL-6) and BMD or greater loss of BMD over time [16], [17]. Evidence has also demonstrated links between inflammatory markers and hip fracture in women [18], [19], [20], osteoporotic fractures in women [21], hip and vertebral fractures in men [22] and non-traumatic fractures in men and women [23].

Therefore, given that adipose tissue distribution is known to differ between ethnic groups, as well as its association with inflammation, we hypothesized that central adiposity or inflammatory status contributed to ethnic differences in BMD in older men and women.

## 2 Material and methods

### 2.1 Participants

The Southall and Brent Revisited Study (SABRE) is a tri-ethnic population-based cohort study consisting of men and women of European, South Asian (originating from the Indian subcontinent) and African or African Caribbean origin. At baseline (1988–1991), two separate studies, the Southall and Brent studies (conducted to the same protocol and by the same team), were established to investigate ethnic differences in cardiometabolic disease; participants living in London and aged 40–69 years were recruited from workplaces or from random selection from general practice lists, and South Asian and African Caribbean participants were first-generation migrants. Further details about baseline studies have been published previously [8]. Subsequently, the baseline studies were combined into the SABRE study, which has undergone two further visits (Visit 2: 2008–2011 and Visit 3: 2014–2018). These analyses are based on cross-sectional data from Visit 3, which included index participants from baseline, partners of index participants and additionally recruited African Caribbean participants. At Visit 3, in addition to measures of cardiometabolic phenotype, participants had dual-energy X-ray absorptiometry (DXA) scans of bone and body composition. Further details of SABRE Visit 3 have also been previously published [24]; ethics approval was granted the London-Fulham NRES Committee and participants gave informed consent.

### 2.2 Anthropometry, biomarkers and questionnaires

The anthropometric measurements included in this study were height, weight and body mass index (BMI). Height was measured using a stadiometer (Seca 216). Weight was measured using a Tanita BC418 body composition analyser.

Blood samples were collected during the clinic visit, approximately 2 h after participants had a light breakfast at home. High sensitivity C-reactive protein (CRP) was measured using an automated platform (c311 Roche Diagnostics, Burgess Hill UK). Interleukin 6 (IL-6) was measured using a high sensitivity ELISA assay (R&D Systems, Biotechne, Oxon UK). All assays used the manufacturers' calibration and quality control material.

In addition, participants were asked to complete three health and lifestyle questionnaires.

### 2.3 Dual-energy X-ray absorptiometry

DXA scans (Hologic, Horizon W (Software version 13.5.3.1), Bedford, MA, USA) of whole body, proximal femur and spine were obtained. Total hip, femoral neck and lumbar spine (L1-L4) BMD (g/cm^2^), as well as fat mass (kg) and lean mass (kg) were measured. The markers of adiposity used in these analyses were derived from DXA body composition measures and included android to gynoid ratio, which is the ratio of percent fat in the android region to percent fat in the gynoid region, and estimated VAT mass (VAT mass). Using the standard definition, lean mass index (LMI) was calculated using total lean mass divided by height^2^.

DXA exclusions were made for extreme outliers (defined as those outside the (1) Upper quartile + (3 × Inter quartile range) or (2) Lower quartile − (3 × Inter quartile range)), poor scan quality and surgery (hip replacement, knee replacement or the presence of pacemakers): femoral neck and total hip BMD were available for 850 participants (of these, 2 exclusions were made), lumbar spine BMD was available for 831 participants (6 exclusions were made) and, from whole body scans, android to gynoid ratio and VAT mass were available for 881 participants (104 exclusions were made) and total lean mass was available for 861 participants (102 exclusions were made).

### 2.4 Statistical analysis

Descriptive statistics were calculated; normally distributed variables were compared between ethnic groups using unpaired *t*-tests and are presented as mean (standard deviation, SD). IL-6 and CRP, which were compared using the Mann-Whitney Rank Sum test, were highly skewed and were therefore log transformed prior to analysis. Sensitivity analyses confirmed the decision to exclude extreme CRP outliers (>30 mg/l) prior to log transformation. Categorical variables were compared using the Chi squared or Fisher's exact test. T-scores were calculated using NHANES references within the manufacturer software and osteoporosis was defined as T-score ≤ −2.5.

Multiple linear regression was used, with age and ethnicity included in all models, and models conducted for men and women separately. The assumption of linearity was visually inspected using scatter plots; no further testing of linearity was conducted. Each of height, BMI, LMI, VAT mass, android to gynoid ratio, logCRP and logIL-6 were added in separate models. In order to avoid introducing multicollinearity between highly correlated predictors, models which included a height-corrected index (BMI, LMI) were not adjusted for height. We chose to add predictors in separate models to distinguish and compare the effects of the potential mechanical and non-mechanical determinants of BMD separately. Pearson's correlation coefficients were used to assess associations between predictors and ensure that multicollinearity was not an issue. Femoral neck, total hip or lumbar spine BMD (g/cm^2^) were chosen for their clinical relevance as sites of osteoporotic fracture and, for clinical relevance, BMD was standardised in the regression models meaning that coefficients represent the difference in BMD, in standard deviations (SD), between ethnic groups. Graphs of the interactions between predictors and ethnicity were plotted based on the estimates from the regression models.

Separate models were repeated with the addition of potential confounders: smoking status, weekly alcohol intake, physical activity (miles of walking per week) and a marker of socioeconomic status (age finished education) and, additionally for women, whether hormone replacement therapy (HRT) had ever been used. Due to missing data, the sample size in the fully adjusted models was reduced; however, all data were used where possible in the main results and sensitivity analyses were used to confirm that descriptive statistics of those with missing confounders (and therefore not included in the fully adjusted models) were similar to the whole population. In men, at the femoral neck and total hip, the maximum sample size was 460 (range: 460–405 depending on the model); this was reduced to 328 (range: 348–312) in the fully adjusted models. At the lumbar spine, the maximum sample size was 443 (range: 443–389), which was reduced to 336 (range: 336–300) in the fully adjusted models. In women, at the femoral neck and total hip, the maximum sample size was 382 (range: 382–313); this was reduced to 256 (range: 256–215) in the fully adjusted models. At the lumbar spine, the maximum sample size was 376 (range: 376–315), which was reduced to 255 (range: 255–219) in the fully adjusted models.

The assumptions of the goodness of fit of the regression models were met. Results are presented as standardised beta coefficient (95% confidence interval); beta is the mean difference in standardised BMD (standard deviations, SD) between ethnic groups. *P*-values <0.05 were considered statistically significant. Analyses were conducted using Stata Version 16 (StataCorp, College Station, Texas, USA).

## 3 Results

### 3.1 Descriptive statistics

A maximum of 483 men (235 European, 173 South Asian and 75 African Caribbean) were included (Table 1). European men were tallest and BMI was lowest in South Asian men. Median CRP levels were higher in European men compared to South Asian men; the difference in IL-6 and CRP levels between African Caribbean and European men were of borderline significance (*p* = 0.05). LMI was highest in African Caribbean men and lowest in South Asian men. VAT mass was highest in European and lowest in African Caribbean men. Smoking status and alcohol intake showed ethnic differences. Based on femoral neck T-score, 5.6% of European men and 5.5% of South Asian men were classed as having osteoporosis; there was an association between T-score and ethnicity (*p* < 0.01).

**Table 1:** Descriptive statistics by ethnic group, for men and women separately.

	Men						Women					
	European		South Asian		African Caribbean		European		South Asian		African Caribbean	
Maximum n	235		173		75		152		115		133	
	Mean	SD	Mean	SD	Mean	SD	Mean	SD	Mean	SD	Mean	SD
Age (years)	75.3	5.3	74.8	4.9	73.8	7.7	72.3^a^, ^b^	6.8	70.5	5.8	69.1	7.4
Height (cm)	172.9^a^, ^b^	6.6	167.6	6.2	171.0^a^	6.1	160.3^a^	5.7	154.4	5.7	160.2^a^	5.5
Weight (kg)	83.3^a^	12.9	72.9	10.6	83.2^a^	13.3	70.9^a^, ^b^	12.2	65.6	11.4	78.5^a^	14.6
BMI (kg/m^2^)	27.8^a^	3.9	25.9	3.3	28.4^a^	4.3	27.7^b^	4.8	27.5	4.4	30.6^a^	5.4
LMI (kg/m^2^)	16.8^a^, ^b^	1.6	15.3	1.5	17.9^a^	1.9	13.9^a^, ^b^	1.4	13.2	1.6	15.3^a^	1.9
VAT mass (kg)^e^	1.02^a^, ^b^	0.34	0.95	0.3	0.85^a^	0.36	0.81	0.34	0.82	0.29	0.77	0.31
Android to gynoid ratio^e^	1.2^b^	0.2	1.3	0.1	1.1^a^	0.2	0.99^a^	0.14	1.03	0.11	0.99^a^	0.14
Femoral neck BMD (g/cm^2^)^c^	0.78^b^	0.14	0.8	0.13	0.92^a^	0.18	0.71^b^	0.11	0.7	0.12	0.81^a^	0.13
Total hip BMD (g/cm^2^)^c^	0.99^b^	0.15	1.0	0.14	1.12^a^	0.19	0.86^b^	0.12	0.87	0.12	0.99^a^	0.12
Lumbar spine BMD (g/cm^2^)^d^	1.09^b^	0.19	1.06	0.2	1.14^a^	0.21	0.93^a^, ^b^	0.15	0.88	0.16	1.02^a^	0.16


	Median	IQR	Median	IQR	Median	IQR	Median	IQR	Median	IQR	Median	IQR
IL-6 (pg/ml)	2.3	1.8–3.4	2.4	1.7–3.7	2.1	1.6–2.8	2.2	1.6–3.1	2.3	1.5–3.9	2.2	1.4–3.0
CRP (mg/l)	1.3^a^	0.6–2.7	1.0	0.5–2.0	1.0	0.5–2.4	1.5	0.6–3.0	1.4	0.7–2.7	1.3	0.6–2.4


	n	%	n	%	n	%	n	%	n	%	n	%
Smoking status⁎												
Never	82	37.6	111	79.9	29	45.3	74	51.4	91	98.9	87	79.1
Ex	129	59.2	25	18	31	48.4	64	44.4	1	1.1	19	17.3
Current	7	3.2	3	2.2	4	6.3	6	4.2	0	0	4	3.6
Alcohol⁎⁎												
<1 unit per week	46	22	77	58.8	30	51.7	49	36	78	90.7	65	67.7
≥1 unit per week	163	78	54	41.2	28	48.3	87	64	8	9.3	31	32.3
Femoral neck T-score⁎⁎												
≥ − 1	90	42.3	84	51.2	48	70.6	56	39.4	34	31.2	86	74.8
−1 to −2.5	111	52.1	71	43.3	20	29.4	72	50.7	63	57.8	28	24.3
≤ − 2.5	12	5.6	9	5.5	0	0	14	9.9	12	11	1	0.9

Abbreviations: BMI: body mass index, LMI: lean mass index: VAT mass: estimated visceral adipose tissue mass, Android:gynoid: android to gynoid ratio (ratio of percent fat in android region to percent fat in gynoid region), CRP: C-reactive protein, IL-6: Interleukin-6.

a Significant difference (*p*-value<0.05) compared to South Asian.

b Significant difference (*p*-value<0.05) compared to African Caribbean.

c Femoral neck and total hip BMD were available for 850 participants (of these, 2 exclusions were made).

d Lumbar spine BMD was available for 831 participants (of these, 6 exclusions were made).

e Android to gynoid ratio and VAT mass were available for 881 participants (of these, 104 exclusions were made).

⁎ Fisher's exact *p*-value < 0.05.

⁎⁎ Chi squared p-value < 0.05.

A maximum of 400 women (152 European, 115 South Asian and 133 African Caribbean) were included in these analyses (Table 1). European and African Caribbean women were taller than South Asian women, whereas BMI was similar, or lower, in South Asian and European women compared to African Caribbean women. LMI was highest in African Caribbean women and lowest in South Asian women. There were no ethnic differences in VAT mass in women. Smoking status and alcohol intake showed ethnic differences. Based on femoral neck T-score, 9.9% of European women, 11.0% of South Asian women and approximately 1% of African Caribbean women were classed as having osteoporosis; there was an association between T-score and ethnicity (*p* < 0.01).

### 3.2 Ethnic differences in BMD

**Fig. 1:**
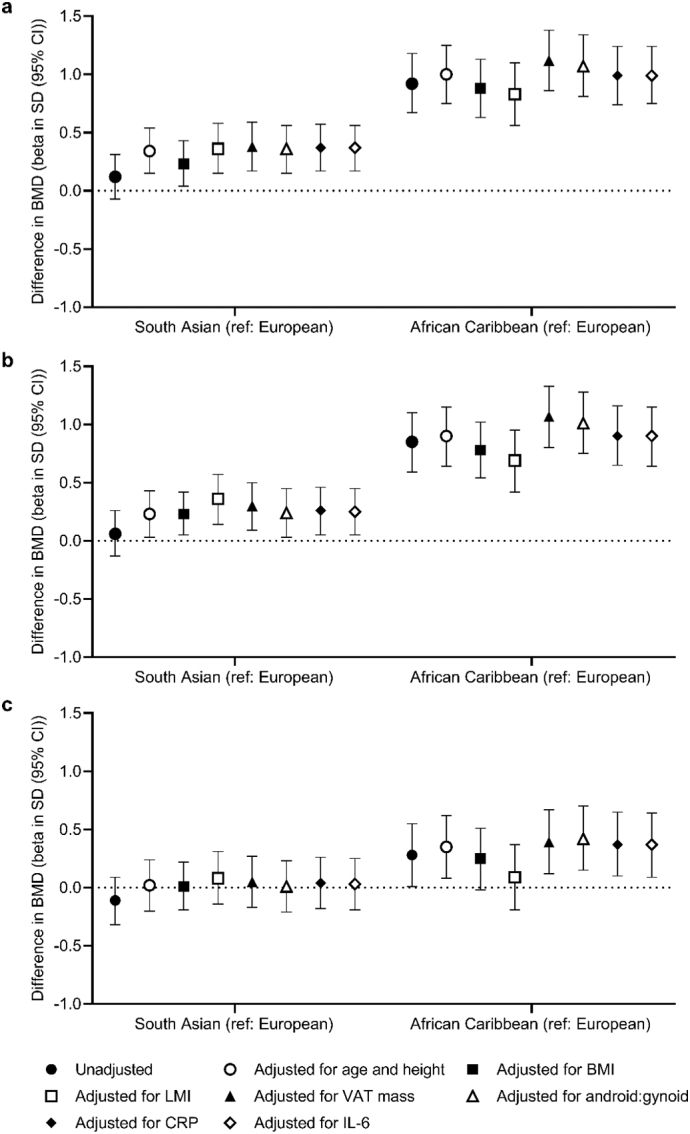
Difference in standardised BMD at the (a) femoral neck, (b) total hip and (c) lumbar spine between ethnic groups (beta in standard deviations (SD) (95% CI)) in men. Except for the unadjusted models: all models were adjusted for age, and models including VAT mass, android:gynoid, CRP and IL-6 were also adjusted for height. Abbreviations: BMI: body mass index, LMI: lean mass index: VAT mass: estimated visceral adipose tissue mass, Android:gynoid: android to gynoid ratio, CRP: C-reactive protein, IL-6: Interleukin-6, ref.: reference ethnic group.

In men, as shown in Figure 1, after adjustment for age and height, the greatest differences in BMD were between African Caribbean and European men (femoral neck: 1.00SD (95% CI: 0.75, 1.25), total hip (0.90SD (95% CI: 0.64, 1.15), lumbar spine: 0.35SD (0.08, 0.62)). There were differences in BMD at the hip between South Asian and European men (femoral neck: 0.34SD (95% CI: 0.15, 0.54), total hip: 0.23SD (95% CI: 0.03, 0.43)), however, there was no difference at the lumbar spine (0.02SD (95% CI: −0.20, 0.24)).

**Fig. 2:**
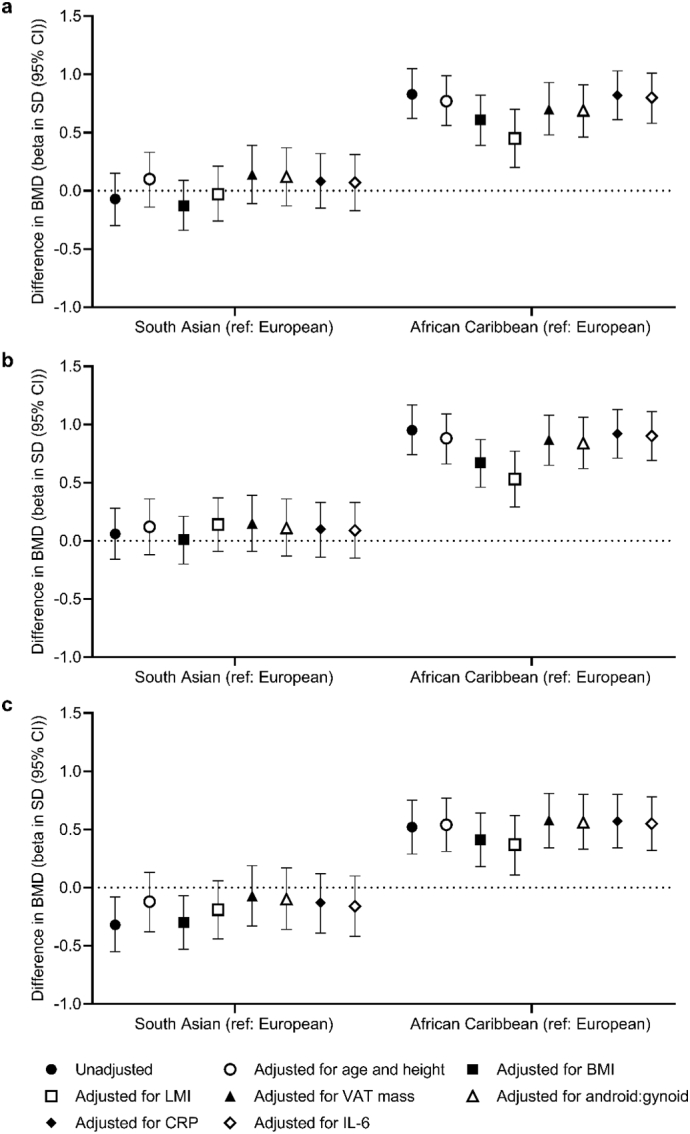
Difference in standardised BMD at the (a) femoral neck, (b) total hip and (c) lumbar spine between ethnic groups (beta in standard deviations (SD) (95% CI)) in women. Except for the unadjusted models: all models were adjusted for age, and models including VAT mass, android:gynoid, CRP and IL-6 were also adjusted for height. Abbreviations: BMI: body mass index, LMI: lean mass index: VAT mass: estimated visceral adipose tissue mass, Android:gynoid: android to gynoid ratio, CRP: C-reactive protein, IL-6: Interleukin-6, ref.: reference ethnic group.

In women, as shown in Figure 2, after adjustment for age and height, BMD was higher in African Caribbean women than European women at all sites (femoral neck: 0.77SD (95% CI: 0.56, 0.99), total hip: 0.88SD (95% CI: 0.66, 1.09), lumbar spine: 0.54SD (95% CI: 0.31, 0.77)). Differences between South Asian and European women did not reach statistical significance (femoral neck: 0.10SD (95% CI: −0.14, 0.33), total hip: 0.12SD (95% CI: −0.12, 0.36), lumbar spine: -0.12SD (95% CI: −0.38, 0.13)).

### 3.3 Are ethnic differences in BMD explained by central adiposity or inflammatory status?

In men, after adjustment for BMI, ethnic differences in femoral neck and total hip BMD remained consistent with the age- and height-adjusted differences (Figure 1 (a) and (b)); however, at the lumbar spine the size of the difference in BMD between African Caribbean and European men (0.35SD (95% CI: 0.08, 0.62)) was reduced (0.25SD (95% CI: −0.02, 0.51)) (Figure 1 (c)). After adjustment for LMI, at the lumbar spine, the size of the difference was further reduced between African Caribbean and European men (0.09SD (95% CI: −0.19, 0.37)). Ethnic differences in BMD were not attenuated by adjustment for VAT mass, android to gynoid ratio, IL-6 or CRP. In separate fully adjusted models, differences in BMD were robust to adjustment for confounders (presented in Supplementary Table S1).

In women, BMD remained higher in African Caribbean women compared to European women at all sites after adjustment for markers of body composition, central adiposity or inflammation (Figure 2). Consistent with the age- and height-adjusted differences, BMD remained similar in South Asian and European women, after further adjustments, at the femoral neck and total hip (Figure 2 (a) and (b)); after adjustment for BMI, lumbar spine BMD was lower in South Asian women (−0.30SD (95% CI: −0.53, −0.07)) (Figure 2 (c)). Ethnic differences in BMD were not explained by adjustment for VAT mass, android to gynoid ratio, IL-6 or CRP. In separate fully adjusted models, the difference in BMD between South Asian and European women at the lumbar spine was attenuated (−0.19SD (95% CI: −0.52, 0.15) (presented in Supplementary Table S2).

### 3.4 Do the relationships between predictors and BMD differ between ethnic groups?

In men, there was no evidence that the relationship between the markers of body composition, central adiposity or inflammatory status and BMD differed between ethnic groups.

**Fig. 3:**
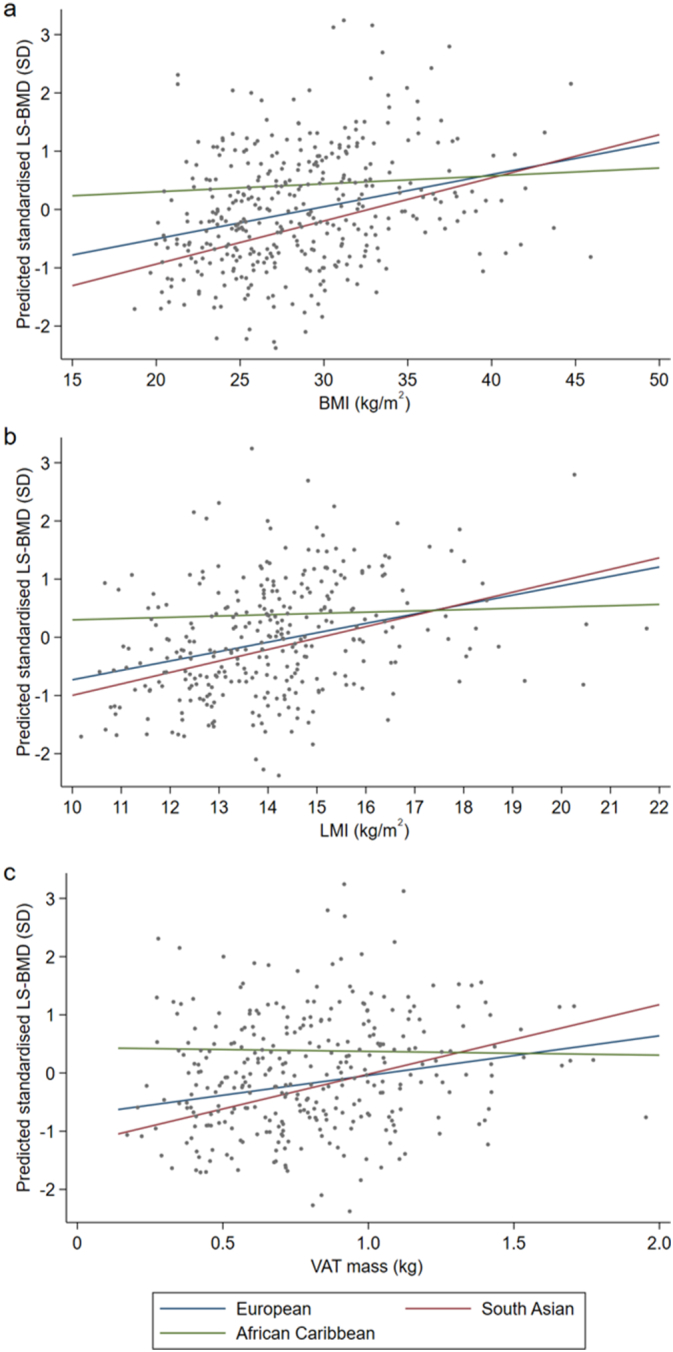
The relationship between (a) BMI, (b) LMI or (c) VAT mass and predicted standardised lumbar spine BMD (SD) in women, with relationships shown for each ethnic group separately. Abbreviations: BMI: body mass index, LMI: lean mass index: VAT mass: estimated visceral adipose tissue mass, LS-BMD: lumbar spine BMD, SD: standard deviation.

In women, with adjustment for age and height, the relationship between BMI, LMI or VAT mass and predicted standardised lumbar spine BMD differed between ethnic groups (overall interactions: BMI: *p* = 0.04, LMI: p = 0.04, VAT mass: *p* = 0.009). Overall, as shown in Figure 3, there was a lack of association between BMI, LMI or VAT mass and lumbar spine BMD in African Caribbean women, compared to a positive association in European and South Asian women. There were no significant differences in these relationships between European and South Asian women. The relationship between VAT mass and lumbar spine BMD differed between European and African Caribbean women (*p* = 0.04).

## 4 Discussion

This study has demonstrated ethnic differences in BMD in SABRE, a tri-ethnic UK-based cohort of older men and women. Contrary to our hypothesis, adjustment for our chosen markers of body composition (BMI and LMI), central adiposity (android to gynoid ratio and VAT mass) or inflammatory status (IL-6 and CRP) did not contribute to the ethnic differences in BMD in SABRE. However, we did find differences in the relationship between markers of body composition (BMI and LMI) and central adiposity (VAT mass) between ethnic groups in women. LMI had the greatest effect on reducing differences in BMD, which is in contrast to the hypothesis that central adiposity and inflammatory markers would contribute to the observed ethnic differences in BMD.

Our data show that BMD was highest in African Caribbean men. Consistent with our results, higher DXA-measured BMD in Afro-Caribbean men compared to European men was previously demonstrated in the European Male Aging Study, and shown to persist at the femoral neck and total hip after adjustment for age, weight and height [4]. In contrast to our study, however, differences between South Asian and White men at the femoral neck and total hip were not statistically significant in the previously described study. Our results are also in agreement with a previous study in which, compared to US Caucasian men, BMD at the femoral neck, total hip and lumbar spine was higher in both Afro-Caribbean (participants of the Tobago Bone Health Study) and African American (participants of the Osteoporotic Fractures in Men Study) men after adjustment for age, weight and height [2].

Our finding that BMD was higher in African Caribbean women compared to European women was similar to previous studies in Afro-Caribbean women (participants of the Tobago Women's Health Study) and African American women (the Study of Osteoporotic Fractures) compared to US Caucasian women [3]. We found similar BMD between South Asian and European women at the hip and, after adjustment for age and height, the lower unadjusted BMD in South Asian women at the spine no longer remained. Previous results have demonstrated lower BMD in South Asian women around the time of peak bone mass; the differences were explained by body size through adjustment for height, weight or BMI [25].

In the current study, at the lumbar spine in men, adjustment for BMI or LMI, reduced the size of the difference in BMD in African Caribbean men compared to European men. Consistent with this finding, lean mass has been demonstrated as a particularly important contributor to the higher bone mineral content in Black men compared to either Hispanic or White men at the femoral neck [26]. Although VAT was previously found to be a predictor of lumbar spine BMD of borderline significance, similar to our findings, it did not explain differences in BMD between men of different ethnic groups, Chinese and Indian men, in a previous study [27].

Finally, the associations between BMI, VAT mass or LMI and lumbar spine BMD differed in women; there was no association between these markers and BMD in African Caribbean women, and positive associations in European and South Asian women. Differences in the relationship between BMI and BMD between post-menopausal Caucasian and African American women have been previously demonstrated and, consistent with our results, a weaker relationship was demonstrated in African American women [28]. Given that we used cross-sectional data, the differences in the relationship between VAT mass, LMI and BMI and lumbar spine BMD which we described reflect differences in the relative proportions of these components of body composition between ethnic groups. Although it was not possible to investigate the longitudinal reasons for these differences in our cohort further, given that our data were cross-sectional, it is possible that these differences could reflect differences in the relative changes in body composition with age between ethnic groups. For example, a greater decline in muscle mass with age has previously been suggested in White women compared to Black women [29].

Given that BMD is a predictor of fracture risk [30], these results contribute further to our understanding of the determinants of ethnic differences in fracture incidence. For example, the higher BMD in African Caribbean men and women in our study, as well as the higher BMD in South Asian men compared to European men, is consistent with previously reported ethnic differences in fracture incidence in men in the UK [1]. Interestingly, in contrast, and in agreement with previous studies, BMD was generally similar in European and South Asian women in SABRE, suggesting the need to consider explanations other than BMD, such as differences in bone microarchitecture, for the lower fracture incidence in South Asian women which has been previously reported [1]. In addition to the use of DXA, it is therefore important to investigate these relationships using other bone imaging techniques such as peripheral quantitative computed tomography (pQCT) and high resolution pQCT, which are able to measure the cortical and trabecular bone compartments separately and other aspects of bone microarchitecture and strength.

A limitation of this study was the relatively small sample size of African Caribbean men, which was the smallest of all ethnic group sizes and it is therefore necessary to consider the effect of this smaller sample size on the power to examine these relationships. In addition, the distribution by ethnicity of individuals with missing confounders, and therefore not included in the fully adjusted models, differed slightly to the proportions in the whole population (men: whole population: 53.70% European, 33.15% South Asian, 13.15% African Caribbean; subgroup with missing confounders: 33.05% European, 44.07% South Asian, 22.88% African Caribbean) (women: whole population: 46.07% European, 23.60% South Asian, 30.34% African Caribbean; subgroup with missing confounders: 21.80% European, 39.10% South Asian, 39.10% African Caribbean). We have not presented results comparing BMD in African Caribbean and South Asian men and women here; in our analyses (not shown) we found very similar patterns to the results which we have presented comparing African Caribbean and European men and women. In addition, we did not adjust our analyses for bone-modifying medications; this was due to the low prevalence of osteoporosis and therefore also the use of bone-modifying medications in our study population. A further limitation was the cross-sectional measurement of BMD, which meant that it was not possible to deduce whether there were differences in the rate of bone loss between ethnic groups, and, in turn, whether this also contributed to the differences in BMD observed. Similarly, it was not possible to determine whether ethnic differences in peak bone mass also contributed to the reported differences in BMD; given that body composition and inflammatory marker data were also cross-sectional, this may have also limited our ability to detect differences in the relationships between the markers of body composition and bone between ethnic groups. Although we used DXA-measured lean mass in our analyses, it is important to note that DXA lean mass is not a direct measure of muscle mass, given that DXA lean mass includes intramuscular fat, fascia and is a projection of muscle tissue, not a physiological cross-section of the muscle. We also did not have measures of muscle force which may also contribute to the observed differences. As such, it is possible that a direct measure of muscle mass, such as D3-creatinine dilution method, or muscle function, may have explained better the differences in BMD between ethnic groups in SABRE. There are limitations to the use of DXA-measured VAT mass compared to other measures of visceral adiposity; there are also limitations to DXA-measured areal BMD, including the fact that bone depth is not taken into account meaning that it is important to account for body size to avoid underestimation of BMD of smaller bones, or overestimation of larger bones. In addition, the bone density is a combined BMD of cortical and trabecular bone meaning compartmental differences cannot be ascertained. Finally, the markers of inflammation and central adiposity which we chose are not exhaustive and may not have been sufficiently sensitive to detect the effect which adiposity and inflammation may have on bone between ethnic groups.

However, a strength of this study was in the use of both DXA-measured body composition parameters (android to gynoid ratio, VAT mass and LMI) in addition to anthropometric measures given that BMI does not provide an indication of relative proportions of fat and lean mass, which are known to differ by ethnicity. A further strength of this study lies in the inclusion of men and women from different ethnic groups in older age, as relatively few studies of well-characterised participants with bone and body composition measurements, which enable the investigation of bone health in older age, exist in the UK.

In conclusion, contrary to our hypothesis, although we observed expected ethnic differences in BMD in later life, in this tri-ethnic UK-based cohort study, we did not find that markers of central adiposity or inflammatory status contributed to these differences. The relationship between ethnicity and bone, which includes lifestyle and environmental influences, as well as genetic variability in osteoporosis risk, is highly complex. Future research is therefore required to further investigate the links between markers of cardiometabolic phenotype, including body composition, adiposity and inflammation, and bone health as populations age and the risk of multiple non-communicable diseases increases.

## CRediT authorship contribution statement

KAW oversaw the work; KAW and RD conceptualised the study. RD prepared the original draft of the manuscript and CP and KAW supervised drafting of the manuscript. RD conducted statistical analysis and CP provided statistical advice. NCH, CC, EMD and KAW contributed musculoskeletal expertise. Clinical expertise, interpretation and resources were provided by TT, SW and NC. All authors contributed to the review of the manuscript.

## Funding

RD is supported by the National Institute for Health Research through the NIHR Southampton Biomedical Research Centre.

The SABRE study was funded at baseline by the Medical Research Council, Diabetes UK, and British Heart Foundation. At follow-up, this research was funded in part by the Wellcome Trust [082464/Z/07/Z] and British Heart Foundation [SP/07/001/23603, PG/08/103, PG/12/29/29497 and CS/13/1/30327]. For the purpose of open access, the author has applied a CC BY public copyright licence to any Author Accepted Manuscript version arising from this submission.

NC receives support from the National Institute for Health Research University College London Hospitals Biomedical Research Centre.

## Role of the funding source

No involvement.

## Declaration of competing interest

NH has received consultancy, lecture fees and honoraria from Alliance for Better Bone Health, AMGEN, MSD, Eli Lilly, Servier, UCS, Shire, Consilient Healthcare, Kyowa Kirin and Internis Pharma outside the submitted work. CC has received consultancy, lecture fees and honoraria from AMGEN, GSK, Alliance for Better Bone Health, MSD, Eli Lilly, Pfizer, Novartis, Servier, Medtronic and Roche outside the submitted work. ED has received consultancy fees from Pfizer, UCB and Lilly. KW has received honoraria from Abbott Nutrition outside the submitted work.

## Appendix A Supplementary data

Supplementary tables Image 1
