# Supplementary material for: Inflammatory status, body composition and ethnic differences in bone mineral density: The Southall and Brent Revisited Study
Source: Bone. 2022 Feb;155:116286. doi: 10.1016/j.bone.2021.116286 (PMC8755916; doi:10.1016/j.bone.2021.116286)
Supplement: Supplementary file 1 — Supplementary tables [file mmc1.docx]

Supplementary Tables

Table S1. Fully adjusted multiple linear regression results for men: the effect of adjustment for body size, body composition, markers of central adiposity and inflammatory status on ethnic differences in standardised BMD at the femoral neck, total hip and lumbar spine

|  | South Asian (reference: European) | | | African Caribbean (reference: European) | | |
| --- | --- | --- | --- | --- | --- | --- |
|  | β | 95% CI | p-value | β | 95% CI | p-value |
| Femoral neck BMD^a^ | | | | | | |
| Age and height | 0.48 | (0.22,0.75) | <0.01 | 1.12 | (0.81,1.43) | <0.01 |
| BMI | 0.32 | (0.06,0.57) | 0.02 | 1.01 | (0.70,1.32) | <0.01 |
| LMI | 0.45 | (0.17,0.73) | <0.01 | 0.80 | (0.47,1.13) | <0.01 |
| VAT mass | 0.48 | (0.20,0.75) | <0.01 | 1.20 | (0.89,1.52) | <0.01 |
| Android:gynoid | 0.47 | (0.19,0.74) | <0.01 | 1.15 | (0.83,1.47) | <0.01 |
| CRP | 0.51 | (0.25,0.77) | <0.01 | 1.10 | (0.79,1.41) | <0.01 |
| IL-6 | 0.51 | (0.25,0.77) | <0.01 | 1.10 | (0.80,1.41) | <0.01 |
| Total hip BMD^a^ | | | | | | |
| Age and height | 0.38 | (0.11,0.64) | 0.01 | 0.99 | (0.68,1.30) | <0.01 |
| BMI | 0.29 | (0.05,0.54) | 0.02 | 0.87 | (0.57,1.17) | <0.01 |
| LMI | 0.44 | (0.16,0.71) | <0.01 | 0.65 | (0.33,0.97) | <0.01 |
| VAT mass | 0.39 | (0.12,0.66) | <0.01 | 1.11 | (0.81,1.42) | <0.01 |
| Android:gynoid | 0.37 | (0.09,0.65) | 0.01 | 1.05 | (0.73,1.37) | <0.01 |
| CRP | 0.39 | (0.13,0.66) | <0.01 | 0.97 | (0.66,1.29) | <0.01 |
| IL-6 | 0.41 | (0.14,0.67) | <0.01 | 0.98 | (0.67,1.29) | <0.01 |
| Lumbar spine BMD^b^ | | | | | | |
| Age and height | 0.15 | (-0.13,0.43) | 0.3 | 0.38 | (0.05,0.71) | 0.03 |
| BMI | 0.08 | (-0.18,0.35) | 0.53 | 0.26 | (-0.06,0.59) | 0.11 |
| LMI | 0.25 | (-0.03,0.54) | 0.08 | 0.13 | (-0.21,0.46) | 0.46 |
| VAT mass | 0.21 | (-0.08,0.50) | 0.15 | 0.48 | (0.15,0.81) | <0.01 |
| Android:gynoid | 0.18 | (-0.11,0.47) | 0.22 | 0.49 | (0.15,0.82) | <0.01 |
| CRP | 0.16 | (-0.12,0.45) | 0.26 | 0.41 | (0.08,0.75) | 0.02 |
| IL-6 | 0.17 | (-0.12,0.45) | 0.25 | 0.40 | (0.06,0.74) | 0.02 |

^a^Maximum sample size in fully adjusted models: 328 (range: 348-312)

^b^Maximum sample size in fully adjusted models: 336 (range: 336-300)

All models were adjusted for age, smoking status (never, ex or current), alcohol intake, age finished education and miles walked per week. Models including VAT mass, android:gynoid, CRP and IL-6 were also adjusted for height.

Abbreviations: BMI: body mass index, LMI: lean mass index: VAT mass: estimated visceral adipose tissue mass, Android:gynoid: android to gynoid ratio (ratio of percent fat in android region to percent fat in gynoid region), CRP: C-reactive protein, IL-6: Interleukin-6.

Table S2. Fully adjusted multiple linear regression results for women: the effect of adjustment for body size, body composition, markers of central adiposity and inflammatory status on ethnic differences in standardised BMD at the femoral neck, total hip and lumbar spine

|  | South Asian (reference: European) | | | African Caribbean (reference: European) | | |
| --- | --- | --- | --- | --- | --- | --- |
|  | β | 95% CI | p-value | β | 95% CI | p-value |
| Femoral neck BMD^a^ | | | | | | |
| Age and height | 0.08 | (-0.24,0.40) | 0.61 | 0.76 | (0.49,1.02) | <0.01 |
| BMI | -0.15 | (-0.43,0.14) | 0.31 | 0.47 | (0.20,0.73) | <0.01 |
| LMI | -0.11 | (-0.42,0.20) | 0.47 | 0.35 | (0.05,0.66) | 0.02 |
| VAT mass | 0.07 | (-0.25,0.39) | 0.67 | 0.72 | (0.44,0.99) | <0.01 |
| Android:gynoid | 0.05 | (-0.28,0.38) | 0.75 | 0.71 | (0.43,0.99) | <0.01 |
| CRP | 0.10 | (-0.22,0.41) | 0.54 | 0.77 | (0.51,1.03) | <0.01 |
| IL-6 | 0.06 | (-0.26,0.38) | 0.69 | 0.74 | (0.47,1.00) | <0.01 |
| Total hip BMD^a^ | | | | | | |
| Age and height | 0.16 | (-0.17,0.49) | 0.34 | 0.88 | (0.61,1.16) | <0.01 |
| BMI | 0.05 | (-0.23,0.34) | 0.71 | 0.57 | (0.31,0.83) | <0.01 |
| LMI | 0.14 | (-0.16,0.44) | 0.35 | 0.48 | (0.19,0.78) | <0.01 |
| VAT mass | 0.16 | (-0.15,0.48) | 0.31 | 0.89 | (0.62,1.16) | <0.01 |
| Android:gynoid | 0.14 | (-0.19,0.46) | 0.4 | 0.88 | (0.61,1.16) | <0.01 |
| CRP | 0.18 | (-0.15,0.50) | 0.29 | 0.90 | (0.63,1.17) | <0.01 |
| IL-6 | 0.15 | (-0.18,0.48) | 0.38 | 0.87 | (0.60,1.14) | <0.01 |
| Lumbar spine BMD^b^ | | | | | | |
| Age and height | 0.001 | (-0.36,0.37) | 0.99 | 0.76 | (0.45,1.06) | <0.01 |
| BMI | -0.19 | (-0.52,0.15) | 0.27 | 0.55 | (0.24,0.85) | <0.01 |
| LMI | -0.12 | (-0.47,0.24) | 0.51 | 0.51 | (0.17,0.85) | <0.01 |
| VAT mass | 0.02 | (-0.35,0.40) | 0.90 | 0.84 | (0.52,1.16) | <0.01 |
| Android:gynoid | 0.01 | (-0.37,0.38) | 0.98 | 0.83 | (0.51,1.16) | <0.01 |
| CRP | 0.01 | (-0.35,0.38) | 0.94 | 0.78 | (0.47,1.08) | <0.01 |
| IL-6 | -0.03 | (-0.39,0.34) | 0.88 | 0.75 | (0.44,1.06) | <0.01 |

^a^Maximum sample size in fully adjusted models: 256 (range: 256-215)

^b^Maximum sample size in fully adjusted models: 255 (range: 255-219)

All models were adjusted for age, smoking status (never, ex or current), alcohol intake, age finished education, miles walked per week and HRT use (ever used HRT yes/no). Models including VAT mass, android:gynoid, CRP and IL-6 were also adjusted for height.

Abbreviations: BMI: body mass index, LMI: lean mass index: VAT mass: estimated visceral adipose tissue mass, Android:gynoid: android to gynoid ratio (ratio of percent fat in android region to percent fat in gynoid region), CRP: C-reactive protein, IL-6: Interleukin-6.
